# PARP Inhibitors in Ovarian Cancer: The Route to “Ithaca”

**DOI:** 10.3390/diagnostics9020055

**Published:** 2019-05-18

**Authors:** Stergios Boussios, Afroditi Karathanasi, Deirdre Cooke, Cherie Neille, Agne Sadauskaite, Michele Moschetta, Nikolaos Zakynthinakis-Kyriakou, Nicholas Pavlidis

**Affiliations:** 1 Acute Oncology Assessment Unit, Medway NHS Foundation Trust, Windmill Road, Gillingham, Kent ME7 5NY, UK; a.karathanasi@nhs.net (A.K.); deirdrecooke@nhs.net (D.C.); cherie.neill@nhs.net (C.N.); 2 AELIA Organization, 9th Km Thessaloniki—Thermi, 57001 Thessaloniki, Greece; 3 Department of Pharmacy, Medway NHS Foundation Trust, Windmill Road, Gillingham, Kent ME7 5NY, UK; agne.sadauskaite@nhs.net; 4 Drug Development Unit, Sarah Cannon Research Institute, 93 Harley Street, London W1G 6AD, UK; michelemoschetta1@gmail.com; 5 Leicester Diabetes Research Centre, Gwendolen Road, Leicester LE5 4PW, UK; nikolaoszakynthinakis@gmail.com; 6 Medical School, University of Ioannina, Stavros Niarchou Avenue, 45110 Ioannina, Greece; npavlid@uoi.gr

**Keywords:** ovarian cancer, *BRCA*, PARP inhibitors, homologous recombination, companion diagnostic, toxic effects, resistance mechanism

## Abstract

Poly (ADP-ribose) polymerase (PARP) inhibitors are a novel class of therapeutic agents that target tumors with deficiencies in the homologous recombination DNA repair pathway. Genomic instability characterizes high-grade serous ovarian cancer (HGSOC), with one half of all tumors displaying defects in the important DNA repair pathway of homologous recombination. Early studies have shown significant efficacy for PARP inhibitors in patients with germline breast related cancer antigens 1 and 2 (*BRCA1/2*) mutations. It has also become evident that *BRCA* wild-type patients with other defects in the homologous recombination repair pathway benefit from this treatment. Companion homologous recombination deficiency (HRD) scores are being developed to guide the selection of patients that are most likely to benefit from PARP inhibition. The choice of which PARP inhibitor is mainly based upon the number of prior therapies and the presence of a *BRCA* mutation or HRD. The identification of patients most likely to benefit from PARP inhibitor therapy in view of HRD and other biomarker assessments is still challenging. The aim of this review is to describe the current evidence for PARP inhibitors in ovarian cancer, their mechanism of action, and the outstanding issues, including the rate of long-term toxicities and the evolution of resistance.

## 1. Introduction

Ovarian cancer is composed of three histological subtypes: epithelial (90%), germ cell (5%), and sex cord stromal cell (5%). Epithelial ovarian cancer (EOC) is the most lethal gynecological disease due to lack of screening test sensitivity [1]. Histologic subtypes of EOC include high- and low-grade serous (75–80%), mucinous (3%), endometrioid (10%), and clear cell (10%) [2]. Typically, EOC is diagnosed being progressed to an advanced stage with the involvement of the peritoneal cavity and other organs [3]. Consequently, the prognosis of the disease is dismal. 

Current first-line treatment of high-grade EOC includes debulking surgery, followed by platinum-based doublet chemotherapy. Although the disease is chemo-sensitive, most patients will eventually experience disease recurrence, following the initial remission. Poly (ADP-ribose) polymerase (PARP) inhibitors have entered into standard treatment for EOC, based on the results from randomized clinical trials demonstrating the significant prolongation of progression-free survival (PFS), accompanied by acceptable tolerability. Olaparib and rucaparib have currently been approved for the treatment of recurrent *BRCA* mutant ovarian cancer, as well as maintenance therapy in platinum-sensitive relapsed disease, whereas niraparib is only indicated for the maintenance setting. Other newer agents, such as talazoparib and veliparib, are in earlier stages of development. In the era of precision medicine, *BRCA* and homologous recombination deficiency (HRD) status represent novel predictive biomarkers of response to chemotherapy and PARP inhibitors [4]. Germline mutations of the genes *BRCA1/2* are related to increased cancer predisposition and they account for approximately 14% of EOCs [5]. These genes encode proteins with a crucial role in the repair of double-strand DNA breaks (DSBs) through HRD. Furthermore, somatic mutations and epigenetic inactivation of *BRCA1/2* have been implicated in sporadic ovarian cancer [6]. Beyond *BRCA1/2*, additional genes are involved in homologous recombination DNA repair. From the clinical point of view, the efficacy of PARP inhibitors includes both germline *BRCA*-mutated ovarian cancer and sporadic ovarian cancers with HRD [7].

It took more than 10 years from the discovery of the synthetic lethality upon PARP inhibition to the regulatory approval of PARP inhibitors. Taken the significant improvement in patients’ benefit observed in earlier therapeutic settings, along with the likelihood of long-term tolerability of PARP inhibitors, there is great potential for this drug class to become a foundation treatment for ovarian cancer, and far beyond *BRCA1/2* mutant tumors.

The purpose of this article is to provide perspective, in the background of various PARP inhibitors for recurrent ovarian cancer treatment, their mechanism of action, tumors’ genomic profiling, accompanied by companion diagnostics, tolerance, and potential resistance mechanisms to PARP inhibitor therapy. 

## 2. PARPs Inhibitors

PARP inhibitors have changed the therapeutic strategy of patients with *BRCA*-related ovarian cancer [8]. These agents have many similarities, but at the same time notable differences, which are based on the differences between their chemical structural features [9]. All of the PARP inhibitors that were developed in EOC are PARP-1 and PARP-2 inhibitors, while olaparib and rucaparib additionally inhibit PARP-3. Furthermore, rucaparib inhibits tankyrase-1, which is another member of the PARP family [10]. Currently, novel agents are in clinical development. Table 1, Table 2, Table 3, Table 4 and Table 5 resume clinical trials of PARPs inhibitors for treatment of ovarian cancer. 

## 3. Olaparib 

Olaparib is the first inhibitor of the PARP enzymes 1, 2, and 3 (PARP-1, PARP-2, and PARP-3 respectively) developed in ovarian cancer. It has been approved by both European Medicines Agency (EMA) and United States (US) Food and Drug Administration (FDA) as maintenance treatment for recurrent EOC, fallopian tube, or primary peritoneal cancer, following complete or partial response to chemotherapy with a platinum compound in patients with somatic or germline mutations in *BRCA1/2*.

Study 19 was a randomized, double-blind, placebo- controlled, phase II study of the use of olaparib as maintenance treatment in the setting of recurrent, platinum sensitive EOC [11]. The patients were eligible, regardless of *BRCA* mutation status, and they should have been treated with two or more prior lines of platinum based therapy with complete or partial response to their most recent platinum regimen. Two hundred sixty-five patients were enrolled and randomized to olaparib 400 mg bid or placebo. Those that were treated with olaparib had a significantly prolonged PFS (8.4 months vs. 4.8 months, hazard ratio (HR) 0.35; 95% confidence interval (CI) 0.25–0.49; *p* < 0.001). This PFS advantage was prominent in all subgroup analyses. A retrospective preplanned analysis of the data by *BRCA* mutation status revealed significant improvement in PFS in patients with either germline, or somatic *BRCA* mutations that were treated with olaparib as compared with placebo (11.2 vs. 4.3 months, HR 0.18; *p* < 0.0001) [12]. However, there is a proportion of patients without a *BRCA* mutation that may also benefit from olaparib (7.4 vs. 5.5 months, HR 0.54; *p* = 0.0075). This PFS advantage was similar in a subgroup analysis of the 10% of patients with a somatic rather than germline *BRCA* mutation (HR 0.23 vs. 0.17 in somatic vs. germline *BRCA* mutations, respectively) [13]. Importantly, olaparib prolonged the time to second subsequent therapy in both *BRCA*-mutated (HR 0.44; *p* < 0.001) and non-*BRCA*-mutated patients (HR 0.64; *p* < 0.34), which suggested that treatment with PARP inhibitors did not affect further response to chemotherapy [12]. This evidence confirmed the hypothesis that HRD and consequent susceptibility to platinum compounds or even other drugs that either create DNA damage, such as pegylated liposomal doxorubicin, or prevent damage repair, like PARP inhibitors, depend on various gene alteration beyond *BRCA* mutation [14]. Following the results of Study 19, the EMA approved olaparib for maintenance treatment of patients with platinum-sensitive relapsed *BRCA*-mutated HGSOC who responded to platinum-based chemotherapy. 

In December 2014, olaparib also received FDA-approval, for use in patients with germline *BRCA*-mutated HGSOC, fallopian tube, or primary peritoneal carcinoma following three or more prior lines of treatment. This was based on the results of Study 42, evaluated olaparib in the dosage of 400 mg bid in heavily pretreated patients with recurrent, platinum resistant ovarian cancer, and a germline *BRCA1/2* mutation, until disease progression or unacceptable toxicity [15]. The overall response rate (ORR) in *BRCA*-mutated ovarian cancer was 34% (range 26%–42%) with a median duration of response of 7.9 months (5.6–9.6 months). 

More recently, the international phase III SOLO2 study evaluated olaparib as maintenance therapy in platinum-sensitive, relapsed ovarian cancer patients with a *BRCA1/2* mutation treated with at least two lines of previous chemotherapy [16]. Two hundred ninety-five patients were randomized to receive 2:1 olaparib tablets 300 mg orally bid or placebo after at least four cycles of platinum based regimens. Maintenance therapy with olaparib demonstrated a dramatic improvement in PFS of 19.1 months versus 5.5 months (HR 0.3; 95% CI 0.22–0.41) in treated patients using 300 mg (two tablets) bid formulation [16]. The results of SOLO2 confirmed Study 19. In view of these data, the FDA recently granted a second therapeutic indication on August 17, 2017, for maintenance following a complete or partial response to platinum-based chemotherapy, regardless *BRCA* mutation status. Subsequently, EMA approved olaparib in maintenance setting in May 2018, based on the multicenter, randomized, double blinded phase III SOLO-1 trial [17]. The final results were presented at the European Society for Medical Oncology 2018 Congress. Four hundred fifty-one *BRCA1/2* mutated patients were randomized in a 2:1 ratio to first-line maintenance with olaparib 300 mg bid or placebo. The median PFS for patients on the placebo arm was only 13.8 months, while the median PFS for those that were treated with olaparib was not reached, but looks to be approximately three years longer than the placebo group (HR 0.30; *p* < 0.0001) [17]. Table 1 summarizes olaparib clinical trial data.

## 4. Niraparib

Niraparib is a potent and selective inhibitor of PARP-1 and PARP-2. It is primarily metabolized by carboxylesterase to form a major inactive metabolite, which subsequently undergoes glucuronidation. The activity and safety of niraparib monotherapy 300 mg OD were initially assessed in the phase I trial, including 42 patients with relapsed ovarian cancer [30]. Pharmacodynamic analyses confirmed that PARP inhibition exceeded 50% at daily doses that were greater than 80 mg and antitumour activity was confirmed beyond doses of 60 mg. The ORR in *BRCA1/2* mutated patients was 40%, and the relevant median duration of response 12.9 months. Interestingly, an ORR of 67% was achieved in platinum-sensitive disease and *BRCA1/2* wild-type patients. These results are compatible with those that were observed in a double-blind, randomized phase III study, investigating the role of niraparib as maintenance therapy in relapsed ovarian cancer [31]. In this trial, 553 EOC patients were randomized 2:1 to orally niraparib 300 mg QD or matched placebo within eight weeks of the most recent therapy, until progression or unacceptable toxicity. All of the patients had been treated with at least two prior platinum-based regimens. The eligible patients were assigned to one of two cohorts; those with germline *BRCA* mutations were assigned to the germline *BRCA* mutated cohort (*n* = 203), whereas patients without germline *BRCA* mutations were assigned to the non-germline *BRCA* mutated cohort (*n* = 350). In the primary efficacy analyses, the *BRCA* wild-type cohort was divided into two subgroups according to HRD status, which were based on companion diagnostic test for HRD. Furthermore, in exploratory analyses, the HRD-positive subgroup was further defined by the presence or a lack of a somatic *BRCA* mutation, respectively (47 patients HRD-positive somatic *BRCA1/2* mutated and 115 patients HRD-positive *BRCA* wild-type). The PFS was independently assessed in the germline *BRCA* and *BRCA* wild-type cohort, and it was found to be longer among the niraparib-treated patients in all groups when compared to the placebo. Among germline *BRCA* mutation carriers, there was a significant prolongation in PFS in the niraparib group as compared to placebo (median PFS 21.0 months vs. 5.5 months, HR 0.27; 95% CI 0.017–0.41; *p* < 0.0001). When the HRD tumors were retrospectively assessed in an exploratory analysis out of the non-germline *BRCA* group, niraparib reduced the risk of progression by 62% (PFS 12.9 months versus 3.8 months; HR 0.38; 95% CI 0.24–0.59). Non-germline *BRCA* mutant and negative HRD patients that were treated with niraparib achieved a smaller, but significant, prolongation of PFS when compared to placebo (9.3 months vs. 3.9 months, HR 0.45; 95% CI 0.34–0.61). Furthermore, PFS was longer in the HRD-positive somatic *BRCA1/2* mutated subgroup, similarly to the germline *BRCA* cohort (20.9 vs. 11.0 months, HR 0.27; *p* = 0.02). In the HRD-positive *BRCA* wild-type subgroup, PFS was 9.3 and 3.7 months in the niraparib group and in the placebo group (HR 0.38; *p* < 0.001), respectively. Finally, in the HRD-negative non-germline *BRCA* mutation subset of patients, the obtained PFS was 6.9 vs. 3.8 months (HR, 0.58; 95% CI 0.36–0.92; *p* = 0.02).

In March 2017, FDA approved niraparib at a dose of 300 mg daily as maintenance treatment of recurrent EOC, fallopian tube, or primary peritoneal cancer, in responders to platinum-based chemotherapy; the approval of niraparib by EMA, at the same context, came in November 2017. Furthermore, in June 2018, the results of the Quadra Trial were presented at the American Society of Clinical Oncology annual meeting [32]. This was a phase II, open-label study of niraparib in the setting of platinum sensitive, HRD-positive, HGSOC. Among the 45 patients treated with three or more previous regimens without prior PARP inhibitor administration, the achieved ORR was 27.5% and the duration of response 9.2 months. Finally, PRIMA (NCT02655016) is an ongoing, phase III, randomized, placebo-controlled study of maintenance niraparib in high-risk patients with HRD advanced ovarian cancer, who responded to first-line platinum-based chemotherapy [33]. Table 2 shows a summary of the trials evaluating niraparib use in the recurrent setting.

## 5. Rucaparib 

Rucaparib is a potent PARP-1 and PARP-2 oral inhibitor, which is approved by FDA in December 2016 and by EMA in May 2018 as monotherapy for the treatment of advanced *BRCA*-mutated ovarian cancer, relapsed after at least two chemotherapy lines. It differs by other PARP inhibitors, because it inhibits tankyrases that promote homologous recombination [34]. Rucaparib is metabolized by *CYP2D6* and, to a lesser extent, by *CYP1A2* and *CYP3A4*. It has been evaluated in two key clinical trials in this setting; the phase I II Study 10 and the phase II trial ARIEL 2. Table 3 summaries additional ongoing clinical trials. 

Part 1 of Study 10 (phase I) included 56 patients with advanced solid tumors and established an optimal dose of 600 mg bid, which is characterized by acceptable toxicities. In Part 2A (phase II), 42 patients with recurrent, platinum-sensitive ovarian cancer, and germline *BRCA1/2* mutations, who were previously treated with two to four lines of chemotherapy, received maintenance rucaparib 600 mg bid [35]. The reported objective response rate was 59.5% and the median duration of response of 7.8 months (range 5.6–10.5). 

The ARIEL 2 study was a multicenter, two-part, phase II open label trial that assessed the efficacy of rucaparib in relapsed HGSOC or endometrioid ovarian carcinoma following one or more (part 1) and three or four prior chemotherapy lines (part 2), independently of the platinum-sensitivity status. ARIEL 2 Part 1 enrolled 192 platinum-sensitive OC patients and stratified the patients into three cohorts [36]; *BRCA1/2*-mutated (*n* = 40), *BRCA* wild-type with loss of heterozygosity (LOH) high (*n* = 82), and *BRCA* wild-type with LOH low (*n* = 70). The median PFS was significantly longer in the *BRCA* mutated subgroup (12.8 months) and in the *BRCA* wild-type LOH high (5.7 months) when compared to *BRCA* wild-type LOH low subgroup (5.2 months). This difference was significant in the *BRCA* mutant group (HR 0.27; 95% CI 0.16–0.44; *p* < 0.0001) when compared to the LOH low group; a similar, though not statistically significant, trend was demonstrated in the LOH high group (HR 0.62; 95% CI 0.42–0.90; *p* = 0.011) as compared to the LOH low group. There was also a notable advantage in the median duration of response in the *BRCA* mutant group (9.2 months) and LOH high group (10.8 months) as compared to the LOH low group (5.6 months), whereas, the ORR was higher in the *BRCA1/2*-mutated (80%) and in the *BRCA* wild-type LOH high (29%) than the *BRCA* wild-type LOH low subgroup (10%). Indeed, this study is the first that prospectively demonstrated that the HRD signature could serve as a predictive biomarker for the PARP inhibitor response in *BRCA* wild-type patients with HGSOC. Interestingly, an analysis of tumor biopsies revealed an association between the methylation of *BRCA1* or *RAD51C* and high LOH, with positive impact on ORR (52.4% and 75%, respectively) and PFS (7.4 months and 11.1 months, respectively). However, the establishment of the predictive value of methylation of *BRCA1* or *RAD51C* requires further study [37]. The second part of ARIEL 2 trial is still ongoing (NCT01891344); 286 patients who have been treated with at least three instances of chemotherapy and recurred with both platinum sensitive or resistant disease have been enrolled with the prospect to of evaluating the clinical activity of rucaparib based on HRD status [38]. Preliminary data incorporating Parts 1 and 2 demonstrate a difference in PFS among *BRCA* mutation carriers on the basis of platinum sensitivity (12.7, 7.3, and 5.0 months in platinum-sensitive, –resistant, and –refractory setting, respectively) [38]. The pooled analysis of the two trials confirmed these encouraging results, including 106 patients with HGSOC and a deleterious *BRCA1/2* mutations [39]. Among them, 42 patients participated in Study 10 (Part 2A) and 64 in both parts of ARIEL 2. All of the patients were treated with at least two prior lines of chemotherapy, while 74.5% exhibited sensitivity to their last platinum-based therapy, 18.8% were platinum resistant, and 8.4% platinum refractory. Germline mutations were detected in 83% all patients, whereas 17% were carriers of somatic alterations. Among them, *BRCA1/2* genes were identified in 63.2 and 36.8%, respectively. The median duration of response was 9.2 months, and no difference in ORR was reported between the *BRCA1/2* mutated subgroups. Additionally, patients with a platinum-free interval that exceeded 12 months had a higher ORR than those with a platinum-free interval of 6–12 months or less than six months. 

ARIEL 3 is a phase III randomized trial of oral rucaparib 600 mg bid versus placebo (2:1 randomization) as maintenance treatment in 564 patients with platinum-sensitive HGSOC or endometrioid ovarian cancer, in response to their recent platinum-based chemotherapy [40]. The HRD signature that was mentioned in ARIEL 2 is also being prospectively assessed in ARIEL 3. The primary endpoint was the PFS, which was evaluated based on the molecular signatures of *BRCA* mutations (germline or somatic), HRD-positivity (including *BRCA* mutant and *BRCA* wild-type with LOH high), and intent-to-treat (all enrolled patients). As such, for BRCA mutated patients, the median PFS was 16.6 months in the rucaparib arm versus 5.4 months in the placebo group (HR 0.23; *p* = 0.0001). Similarly, in BRCA wild-type patients, the reported PFS for those with an HRD-positive disease treated with rucaparib was 13.6 months as compared to 5.4 for the placebo group (HR 0.32; *p* = 0.0001), while, in the intention to treat population, it was 10.8 versus 5.4 months, respectively (HR 0.36; *p* = 0.0001). The secondary endpoint of blinded independent central review PFS was also significant for each molecular signature separately: germline *BRCA* mutation (26.8 months vs. 5.4 months), homologous recombination deficient high LOH (22.9 months versus 5.5 months), and overall intent-to-treat populations (13.7 months vs. 5.4 months). An exploratory analysis in the *BRCA* wild-type only revealed a maintained benefit of rucaparib in both the HRD-positive (median PFS 9.7 months, HR 0.44; *p* < 0.0001) and HRD-negative subsets (median PFS 6.7 months, HR 0.58; *p* = 0.0049). 

Finally, ARIEL 4 (NCT02855944) is an ongoing phase III study, which was designed to compare the efficacy and the safety of rucaparib versus physician’s choice of chemotherapy, depending on platinum status, in *BRCA1/2* mutated recurrent ovarian cancer following at least two previous lines of systemic treatment [41]. Primary endpoint is PFS with a target enrollment of 345 patients. Rucaparib is also being explored in combination with programmed death-ligand 1 (PD-L1) inhibitor atezolizumab in a phase Ib trial of patients with recurrent, platinum-sensitive ovarian cancer (NCT03101280) [42]. Table 3 lists the randomized studies of rucaparib in ovarian cancer. 

## 6. Veliparib 

Veliparib is a potent inhibitor of PARP1/2, which was demonstrated in 2007 to have high anti-tumor efficacy when combined with DNA alkylating agents, such as temozolomide and irradiation [43]. Even though evidence supporting the use of veliparib in the treatment of EOC is limited as compared to other PARP inhibitors, there are still several ongoing studies, with veliparib, either as monotherapy, or, combined with chemotherapy. 

In GOG 280, a phase II, single-arm trial, veliparib 400 mg bid was administered to 50 *BRCA* mutated patients with persistent or recurrent ovarian cancer, which were previously treated with up to three prior lines of chemotherapy, until progression or intolerance [44]. The ORR to veliparib was 26% (90% CI: 16–38%); nevertheless, 31 out of 50 patients (61%) progressed on treatment, whereas response in the platinum-resistant and platinum-sensitive patients was 20 and 35%, respectively, with a median PFS of 8.2 months (ranging from 0.43 to 19.55 months) and a median OS of 19.7 months (ranging from 2.3 to 19.7 months). A phase I trial evaluated the maximum tolerated dose, pharmacokinetic and pharmacodynamics properties, and clinical response of veliparib [45]. The recommended phase 2 dose was 400 mg bid, whereas the ORR in *BRCA* mutated, and *BRCA* wild-type patients was 23% and 4%, respectively, accompanied by a clinical benefit rate of 58% and 38%, across all dose levels. A more recent phase I II trial assessed the role of single agent veliparib in patients with germline *BRCA* mutations, in the setting of platinum sensitive or resistant ovarian cancer. Sixteen participants were enrolled in the phase I study and 32 in the phase II. The maximum tolerated dose was established at 300 mg twice daily. Median PFS and overall survival (OS) for the intention to treat population were 5.6 and 13.7 months, respectively [46]. 

A phase II trial randomized 72 patients with *BRCA*-mutated ovarian cancer to oral cyclophosphamide 50 mg daily with or without veliparib 60 mg PO daily. The combination of veliparib with cyclophosphamide did not improve ORR (11.8% vs. 19.4%, respectively) or median PFS (2.1 months vs. 2.3 months, respectively; *p* = 0.68) as compared to single agent cyclophosphamide [47]. Similarly, no responses were reported in phase I study of veliparib in combination with topotecan [48]. GOG 3005, a double-blind, randomized phase III trial for the evaluation of veliparib as first-line treatment in association with carboplatin and paclitaxel in newly diagnosed patients with stage III IV EOC is currently recruiting participants (*n* = 1140; NCT02470585) [49]. The study includes three treatment arms: chemotherapy only, chemotherapy followed by veliparib switch maintenance, and veliparib combined with chemotherapy followed by continuation maintenance. 

In a phase I trial, Reiss et al. evaluated the activity of veliparib combined with low-dose fractionated whole abdominal radiation therapy in 32 patients with peritoneal carcinomatosis due to advanced solid tumors. Among them, 18 were patients with EOC, five of whom with *BRCA* mutations. The reported ORR was only a 3%, while a stable disease was exhibited by 33% of patients. Those with *BRCA* mutated EOC had a PFS of 4.47 months, as compared to 3.58 months for the *BRCA* wild-type cohort and an OS of 10.15 months versus 7.89 months, respectively. The PFS of the patients with platinum-sensitive disease was 7.92 months, while those with platinum-resistant EOC reached 3.58 months [50]. Table 4 depicts the completed or ongoing randomized studies testing veliparib in recurrent and maintenance setting. 

## 7. Talazoparib

Talazoparib is a PARP1/2 inhibitor, which is selective against *BRCA1/2* and phosphatase and tensin homolog (*PTEN*) mutants, enhances the cytotoxic activity of temozolomide, SN-38, and carboplatin [51]. The efficacy of talazoparib in the treatment of ovarian cancer is still under investigation. It initially assessed in a two-stage dose-escalation trial study, with the enrolment of 34 EOC patients, of whom 25 with germline *BRCA* mutations, all being previously treated with platinum-based chemotherapy [52]. The reported ORR was 42% and median PFS 36.4 weeks in a well-tolerated dose of 1000 μg day. More recently, a phase I study evaluated talazoparib in combination with carboplatin in patients with several solid tumors independently of germline status. Among the 24 participants, two had EOC (8%), while 20% identified *BRCA1/2* mutations. In the subset of *BRCA* mutated patients, 1 and 2 cases achieved complete and partial responses, respectively, whereas three patients with somatic *BRCA* mutation maintained a stable disease beyond four months [53]. 

The benefit of talazoparib has been established in a phase II ABRAZO study in terms of patients specifically with *BRCA* mutations. Enrolment was restricted to patients with breast cancer and the reported ORR for *BRCA1* and *BRCA2* mutations carriers was 24% and 34%, respectively [54]. However, data from phase II or III studies in patients with EOC are still not available. Nevertheless, a phase II trial of talazoparib in recurrent *BRCA1/2*-mutated ovarian cancer patients, following primary progression on prior PARP inhibitor, has recently been completed (NCT02326844) [55]. It will be interesting to be clarified whether there is a role for further PARP inhibition in this context. 

Currently, there are several trials actively recruiting patients evaluating talazoparib either as monotherapy or in combination. With this regard, a phase 1 study assesses the combination with a checkpoint inhibitor (NCT03330405) [56], whereas, a phase II study exploring talazoparib activity in advanced cancers with PTEN mutations or *PTEN* loss and HRD defect (NCT02286687) [57]. Furthermore, an ongoing phase II randomized study (NCT02836028) evaluates the activity and tolerance of single agent talazoparib versus combination with temozolomide in patients with *BRCA*-mutated or homologous recombination-deficient relapsed ovarian cancer [58]. Table 5 lists studies of talazoparib in ovarian cancer that have been completed or are currently ongoing.

## 8. Functional Aspects of PARP1

PARP1 initiates and modulates multiple DNA repair pathways, and it is thus important for maintaining genomic integrity. Transcriptional regulation by PARP1 involves both ADP-ribosylation-dependent and independent mechanisms. PARP1 might also regulate transcription by modulating the chromatin structure, altering DNA methylation patterns, acting as a co-regulator of transcription factors, and interacting with chromatin insulators [61]. Under physiological conditions, PARP1 ADP-ribosylation activity curiously follows the rhythmic circadian cycle [62]. 

The interaction between PARP1 and the NF-κB pathway promotes the production of several pro-inflammatory cytokines, including TNFα, IL-6, INFγ, E-selectin, and ICAM-1 [63]; PARP inhibition attenuates the upregulation of these factors in response to inflammatory stimuli, and in parallel prevents inflammation-associated side effects of cytotoxics [64]. The loss of PARP1 activity inhibits proliferative signaling and metastasis through anti-inflammatory mechanisms [65,66].

PARP1 also regulates the c-Jun N-terminal kinase (JNK) pathway, which is implicated as a driver of both tumor development and treatment response [67]. PARP1 downregulates MAP kinase phosphatase MKP-1 expression and inhibits the survival kinase Akt, both of which activate JNK [68]. Based on that, PARP inhibition could be potentially therapeutically beneficial in ovarian cancer taken the elevated JNK activity. PARP1 inhibitors promote Akt activity and mTOR signaling, which leads to decreased cell death [69].

In addition to the JNK-mediated signaling, extracellular signal-regulated kinases (ERKs) represent a second family of MAP kinases that participate in cell death determination, tumor progression, angiogenesis, and metastasis. ERK activation is pivotal in cancer cell survival through the upregulation of anti-apoptotic proteins and inhibition of caspase activity [70]. The jnhibition of this pathway by targeting ERK or MEK leads to suppression of ovarian tumor growth [71]. Indeed, PARP1 inhibition causes a loss of ERK2 stimulation by decreasing the activity of critical pro-angiogenic factors, including vascular endothelial growth factor (*VEGF*) and hypoxia inducible factor (*HIF*).

## 9. PARP and Immune-Checkpoint Inhibitor Combinations

PARP has a well-established proinflammatory role and, in preclinical models, PARP inhibitors attenuate chronic inflammatory and autoimmune conditions [72]. Furthermore, conventional and targeted anticancer therapeutic approaches affect tumor-targeting immune responses. Indeed, the identification of crosstalk between cytotoxic anticancer agents and cancer-associated immunity may increase the effectiveness of combination strategies [73]. PARP inhibition has been shown to lead to the upregulation of PD-L, both in vivo and in vitro. An intrinsic upregulation of PD-L1 may function to inhibit immune responses downstream of PARP inhibitor–mediated priming, and could potentially be overcome through the combination of PARP inhibitor and PD-L1 blockade [74].

In HGSOC, tumors harboring *BRCA1/2* mutations demonstrated a higher neoantigen burden, as well as CD3+ and CD8+ tumor-infiltrating lymphocytes. Increased levels of PD-1 and PD-L1 expression on tumor-infiltrating immune cells as compared to homologous recombination proficient tumors indicates that PD-1/PD-L1 inhibitors may be more effective in *BRCA1/2*-mutated rather than in the homologous recombination proficient HGSOC [75].

Currently, there are available data from three different PARP inhibitor anti–PD-1/L1 combinations. Olaparib durvalumab, and niraparib pembrolizumab combinations were well tolerated, with toxicities that are in line with those that were observed for the relevant agents in monotherapy settings [76,77,78]. In contrast, tislelizumab pamiparib combination demonstrated an increased rate of hepatic toxicity, which suggests that the tolerability of PARP inhibitor anti–PD-1 L1 combinations is variable [79]. In platinum-resistant ovarian cancer the niraparib pembrolizumab combination demonstrated an ORR of 25% [78]; this response rate is similar to that of the PARP inhibitor monotherapy in *BRCA1/2*-mutant patients in this setting [80]. However, it is impressive that the activity of the combination was independent of the DNA damage repair defect status. In relapsed platinum-sensitive disease, *BRCA1/2*-mutant ovarian cancer, the olaparib durvalumab combination demonstrated an ORR of 63%, which is also compatible with the PARP inhibitor monotherapy activity in this setting [80].

## 10. Potential Homologous Recombination Pathway Targets 

DNA damage is often during cell cycle, and it can result in either single-strand DNA breaks or DSBs. Consequently, the loss of genome integrity and cell death are occurred if DNA is not correctly repaired [81]. The success of the inhibition of DNA repair pathways in therapy relies on the identification of inhibitors that target repair proteins that are either directly involved in tumorigenesis or have a synthetic lethal relationship with other repair genes [82]. The inhibition of PARP proteins radiosensitizes glioma cells by inhibiting DNA repair. Studies show that PARP inhibitors decreased the colony formation in *O-6-methylguanine-DNAmethyltransferase* (*MGMT*) unmethylated glioblastoma multiforme patient derived xenografts. Radiotherapy in glioblastoma multiforme patients’ lead to the upregulation of PARP1 mediated repair of DNA damage in glioblastoma cancer stem cells [83]. Furthermore, synthetic lethality is a situation that arises when two nonlethal defects combine and result in cell death [84].

Homologous recombination and non-Homologous End Joining (NHEJ) are the predominant DSBs repair pathways. Homologous recombination is a precise repair mechanism, which is active during phase S-G2 of the cell cycle, which mediates to limit genetic instability [85]. NHEJ is faster than homologous recombination; nevertheless, it is prone to error [86,87]. Alternative NHEJ (alt-NHEJ) was originally identified as a backup repair mechanism in the absence of classical NHEJ factors, but recent studies have demonstrated that alt-NHEJ is active, even when NHEJ as well as homologous recombination is available.

Microhomology-mediated end-joining (MMEJ) is a subtype of alt-NHEJ in the G1-phase [88]. Dissecting the mechanisms of MMEJ is of great interest, because of its potential to destabilize the genome through gene deletions and chromosomal rearrangements in cells that are deficient in canonical repair pathways, including HR and C-NHEJ. The low fidelity DNA polymerase Polθ [89], which is encoded by POLQ, is capable of extending the mismatched termini, ssDNA, and partial ssDNA [90]. Polθ and its fly ortholog dmMus308 have been implicated in the MMEJ of eroded telomeres and DSBs induced by stressed replication forks [91]. In budding yeast, there is no Polθ homologue; instead, polymerase delta (Polδ) and Pol4 are important in yeast MMEJ [92]. A recent study showed that *BRCA*2-deficient ovarian cancer cells express high levels of POLQ, which may contribute to elevated MMEJ in these cells [93]. Therefore, the inhibition of Polθ sensitizes these cells to genotoxic chemicals and PARP1 inhibitor treatment, which indicates Polθ inhibition as a novel cancer therapeutic strategy.

The first studied homologous recombination proteins were the *BRCA1* and *2* [94,95]. Their germline mutations and, consequently, loss of function were associated with the higher risk of breast (ranging from 57% in *BRCA1* mutation to 45% in *BRCA2* mutation) and ovarian cancers (ranging from 11% in *BRCA1* mutation to 40% in *BRCA2* mutation) [96]. Homologous recombination-deficient cancers are potentially sensitive to pharmaceutical agents that induce lesions, which are normally repaired by the homologous recombination pathway. The PARP inhibitors are synthetically lethal in homologous recombination-deficient cells, such as *BRCA1/2*-mutated tumors [97].

Furthermore, the use of PARP inhibitors following the principles of synthetic lethality may be utilized beyond germline *BRCA*-mutated ovarian cancer in the context of HRD. The term “*BRCA*ness” describes tumors that have not arisen from a germline *BRCA1/2* mutation, but, despite that, share certain phenotypes [98]. Several efforts have been made to standardize a test that can optimally identify HRD EOCs. With this regard, transcriptional and mutational signature profiling, proteomics [99] and RNA analyses [100] may identify *BRCA*ness and PARP inhibitor sensitive tumors [98]. In addition, these methods may evaluate the predictive role of prior platinum sensitivity for *BRCA*ness and the subsequent PARP inhibitor responses [18,55]. The limitations of HRD testing include prioritization and the predicted effect of observed mutations, with little emphasis on gene silencing via DNA methylation and false positive results. Increased DNA damage not only promotes immune priming through molecular mechanisms, but it also leads to the adaptive upregulation of PD-L1 expression [74].

Beyond *BRCA1/2* mutant cells that are highly susceptible to PARP inhibitors, deficiencies in a number of tumour suppressor genes participated in homologous recombination repair, such as *ATM ATR* (Ataxia-telangiectasia mutated and *ATM* and *RAD3*-related), *PALB2*, *CHEK2*, *BARD1*, *BRIP1*, *MRE11*, *RAD50*, *NBS1*, *RAD51C*, *RAD51D*, and the *FANC* gene family, were also displayed to confer sensitivity to these drugs [98]. With this regard, the accumulation of *RAD51* at the DNA lesion is an established marker of homologous recombination proficiency; consequently, its absence following DNA damage has been served as a functional biomarker of homologous recombination dysfunction [101]. As such, the detection of *RAD51* foci by immunohistochemistry, demonstrates the predictive factor of response to chemotherapy and PARP inhibition [102]. Many rare diseases are caused by the altered function of DNA damage response genes causing genome instability. Mutations in these genes may have downstream cancer burden that is associated with them. Hence, understanding the biology of rare DNA repair diseases can help to identify novel cancer therapies [103]. The Fanconi Anemia signaling network contains a unique nuclear protein complex that mediates the monoubiquitylation of the *FANCD2* and *FANCI* heterodimer, and coordinates the activities of the downstream DNA repair pathway, including nucleotide excision repair, translesion synthesis, and homologous recombination [104]. In addition, the homozygous deletion of *PTEN* in 7% of EOC is proposed to downregulate *RAD51*, which lead to synthetic lethality upon PARP inhibition [14]. Finally, the amplification of the 11q13 locus resulted in the overexpression of *EMSY*, which is a suppressor of *BRCA2* transcriptional activity in 14% of EOCs [105].

## 11. Combining PARP Inhibition with Companion Diagnostics 

A comprehensive understanding of companion diagnostics for PARP inhibitors is a step forward to personalized treatment, which is based on the power of whole-genome analysis. Companion diagnostics for PARP inhibitors permit optimization of patients’ selection with the greatest chance of achieving a response, either in maintenance or recurrent settings. 

Currently, there are several available FDA-approved companion diagnostic tests for PARP inhibitors. *BRAC*Analysis CDx consists of two in vitro assays for germline *BRCA1/2* mutational identification; *BRAC*Analysis CDx Sanger sequencing; and, *BRAC*Analysis CDx Large Rearrangement Test (BART^®^) for sequence variants, and large rearrangements, respectively. Polymerase chain reaction (PCR) and subsequent Sanger sequencing assess exons and exon intron boundaries of *BRCA1/2* for single nucleotide polymorphism, insertions ≤2 base pairs (bp), and deletions ≤5 bp. On the other hand, BART^®^ identifies single- and multi-exon deletions duplications, flanking introns, the Portuguese founder mutation, and proximal promoter sequences, based on multiplex PCR. Variants that were identified by *BRAC*Analysis CDx require confirmatory analysis by either alternate primer sequencing or PCR analysis, at approximately 1–2%. *BRAC*Analysis CDx limitations include the detection of deletions >5 bp, insertions >2 bp, RNA transcript processing errors, and finally differentiation between gene duplication and triplication [106]. 

Myriad’s myChoice HRD is an enhancement of *BRAC*Analysis CDx, taken that it detects both germline and somatic *BRCA1/2* mutations together with HRD through its evaluation of genomic scarring. This NGS-based assay creates a genomic scarring composite score (HRD Score), which is a sum of LOH, telomeric allelic imbalance (TAI), and large-scale state transitions (LST). The LOH regions are ≥15 Mb, but are shorter than chromosomal length [107], whereas TAI determines regions with allelic imbalance that extend to the subtelomere, without crossing the centromere [108]. Taken the confirmed inverse proportion between *BRCA1* levels and the number of TAI regions in *BRCA1/2* wild-type HGSOC, a high TAI score potentially designates DNA repair defects. Finally, LST evaluates the chromosomal breaks in adjacent regions ≥10 Mb after filtering all variation ≤3 Mb [109]. The tumors are characterized with a score on a scale of 0–100, and patients with scores ≥42 are considered to have high HRD [110]. In NOVA study, among the 174 analyzed tumor samples, myChoice HRD identified 100% (68 68) of germline *BRCA* mutated tumors, and 57% (61 106) of germline *BRCA* wild-type patients with HRD [111]. Based on that, myChoice HRD has been approved by FDA to stratify the germline *BRCA1/2* patients into homologous recombination deficient or proficient cohorts [31]. 

In contrast to *BRAC*Analysis CDx, FoundationOneTM utilizes archival formalin-fixed, paraffin-embedded solid tumor samples, and represents the first FDA approved comprehensive genomic profiling analysis for all solid tumours. It involves the parallel DNA sequencing of a panel of 324 genes to detect genomic alterations that may be therapeutically targetable. *BRCA1/2*, *PALB2*, *FANCM*, *BARD1*, *CHK1*, *ATM*, *RAD51C*, *RAD51B*, and *BLM* are genes that could be potentially targeted by the PARP inhibitor [112]. ARIEL 2 and ARIEL 3 trials evaluated the percentage of genomic LOH in tumour samples using the FoundationOne [36,40]. ARIEL 2 utilized a cutoff greater than 14% to define high LOH; the relevant cutoff that was used by ARIEL 3 was greater than 16%, based on the analysis of LOH from ARIEL 2. Interestingly, in ARIEL 2 trial, BRCA wild-type patients with LOH had improved ORR as compared to those without LOH [44% (95% CI 33–55%) versus 20% (95% CI 11–31%), respectively] [36]. This was not the case in ARIEL 3 trial, which demonstrated that LOH status did not alter the clinical benefit in the subset of *BRCA* wild-type patients [40]. The urgent need for further research in order to be clarified the impact of LOH on PARP inhibitor response is obvious.

## 12. Safety and Tolerability 

The synthetic lethality mechanism of action may have protective effect against severe PARP inhibitor toxicity. However, the consideration of PARP inhibitors induced toxicity is critical, especially in patients that are being treated with multiple lines of chemotherapy. The most common side effects include gastro-intestinal manifestations, myelosuppression, and fatigue. Table 6 depicts the adverse events (AEs) in patients treated with FDA-EMA approved PARP inhibitors.

Gastrointestinal AEs are manageable with dose modification and symptomatic treatment, similarly to the management of chemotherapy-induced gastrointestinal toxicities [113]. In Study 19, the majority of patients experienced at least one AE, mostly grade 1 2, with nausea (68% vs. 35%) and vomiting (32% vs. 14%) being more frequent in the olaparib group. Grade 3 4 AEs were exhibited in 35.3, and 20.3% of the olaparib and placebo group, respectively, which led to more frequent dose interruptions (27.9% vs. 8.6%) and reductions (22.8% vs. 4.7%) in the olaparib arm of the study [11]. AEs mostly occurred within the period between the fourth and eighth week following treatment, and they were generally transient. Similarly, nausea, and vomiting were the most common gastrointestinal AEs in Study 42, occurring in 62, and 39% of patients, respectively [15]. In addition, grade 3 4 toxicities were more frequent when compared to Study 19, occurring in 54% of patients, and they resulted in dose interruption or reduction in 40% of them. The relevant specific patient populations and treatment indications of each study may explain the different rates of AEs. Indeed, study 19 evaluated maintenance treatment in the setting of platinum-sensitive disease, whereas study 42 the treatment of advanced ovarian cancer in platinum-resistant recurrence. Daily prokinetic and antihistamine (e.g., 5-HT3) drugs are generally effective in the symptom control of nausea. Several antiemetics are available, including metoclopramide, prochlorperazine, phenothiazine, dexamethasone, olanzapine, haloperidol, or lorazepam. On the other hand, neurokinin-1 receptor antagonist aprepitant is a strong *CYP3A4* inhibitor that may affect olaparib plasma concentrations, and it is better to be avoided [114]. Additional gastrointestinal symptoms include constipation and diarrhea, treated with senna or polyethylene glycol 3350, and loperamide, respectively. Furthermore, among the three PARP inhibitors, olaparib is associated with the most grade 3 or 4 events of abdominal pain (3%); however, the differentiation of other causes that are potentially related to the underlying malignancy should be made [115]. Finally, dyspepsia and dysgeusia are more frequent in patients that were treated with rucaparib, as compared to olaparib or niraparib; nevertheless, severe toxicity is rare (<1%). The symptomatic management includes proton pump inhibitors that are accompanied by prokinetics, and measures for improvement in oral hygiene, respectively. 

Haematological toxicities usually occur early following treatment initiation and recovery is reliable a few months later. Anaemia was more pronounced with olaparib and rucaparib until the fifth or sixth cycle, and then plateaus with continued treatment [16,40]. In the three available phase 3 maintenance trials, all-grade anaemia has been reported in 44, 50, and 37% of patients who received olaparib, niraparib, and rucaparib, respectively [16,31,40]. Grade 3 4 AEs were slightly higher for the niraparib treated patients (25%), followed by the cohorts of rucaparib and olaparib (19% for each subset). 

Thrombocytopenia of any grade has been described in 61% of patients that were treated with niraparib, as compared to 28 and 14% in those that received rucaparib and olaparib, respectively. The relevant grade 3 4 AEs occurred in 34, 5, and 1% in the niraparib, rucaparib, and olaparib cohort patients, respectively [16,31,40]. With this regard, FDA recommends weekly platelet count monitoring during the first month of treatment with niraparib. There is evidence that platelets and baseline bodyweight could serve as predictors of dose adjustments in patients that were treated with niraparib at 300 mg OD [116]. Those with platelet counts of less than 15 × 10^4^ cells mL, or a baseline bodyweight of less than 77 kg are at higher risk of grade 3 4 thrombocytopenia during the first month (35% vs. 12%), and may have better tolerance with a starting dose of 200 mg OD. 

The development of acute myeloid leukemia (AML) and myelodysplastic syndrome (MDS) in patients that were treated with olaparib is unusual. In Study 19, 2.2% of patients in the olaparib arm developed AML MDS, versus 0.8% in the placebo arm [12]. Study 42 demonstrated that patients on olaparib arm, who were heavily pre-treated, developed MDS AML. It seems that long term follow up is important in the clarification of this issue [15]. 

The PARP inhibitors may also elevate creatinine concentrations; nevertheless, this might not always affect the glomerular filtration rate or lead to renal failure. Rucaparib inhibits kidney transporter proteins *MATE1* and *MATE2-K*, which affect the secretion of creatinine [40]. The ARIEL 3 trial demonstrated an increase of creatinine in 15% of patients in the rucaparib arm, as compared to 2% in the placebo. Veliparib additionally interacts with the primarily renal uptake transporter *OCT2*. SOLO2 trial reported grade 1 2 elevation in creatinine in 11% of patients that were treated with olaparib, versus 1% in the placebo group [16]. On the other hand, niraparib was not related to elevated serum creatinine. Overall, dose reductions or interruptions of PARP inhibitor can be avoided if the glomerular filtration rate is not affected; its assessment should be based on radionucleotide scan. 

PARP inhibitors are also associated with hypercholesterolemia and hypertransaminasemia. Rucaparib increased cholesterol levels of any grade in 40–84% of patients; nevertheless, serious AEs of grade 3 4 hypercholesterolemia were only reported in 2–4% of patients [36]. The suggested treatment is the initiation of statins with the regular monitoring of liver enzymes [117]. ARIEL 3 demonstrated that 34% of rucaparib treated patients developed hypertransaminasemia of any grade, which, however, is overall transient. There are also reports for elevation of alanine aminotransferase (*ALT*) and aspartate aminotransferase (*AST*) in 36 and 28% of patients that were treated with niraparib, respectively. In contrast, olaparib is better tolerated with reported incidences of increased *ALT* and *AST* of 5 and 2%, respectively. 

## 13. Resistance 

Advances in the understanding of PARP inhibitors’ resistance is of paramount importance, and it may lead to novel insights into basic mechanisms of the DNA damage response. Each PARP inhibitor has separate chemical structure with diverse off-target effects [118]. This indicates that the utilization of a secondary PARP inhibitor could be therapeutically beneficial in a resistant tumor. The restoration of homology-directed DNA repair through secondary reversion mutations is the most common identified mechanism of resistance [119]. Indeed, the restoration of *BRCA* activity starts from *BRCA*-deficient and chemo-sensitive cells as a result of several mutations that are induced by platinum agents. This initial restored clone expands in the setting of treatment-specific selective pressure [120]. In this context, somatic *BRCA1/2* mutations were predicted to restore the protein function in the germline *BRCA1/2* mutated ovarian cancer patients in a study [121]. Among 46 women that were exposed to tumor sequencing, 28% (13 out of 46; 95% CI 17.3–42.6%) possessed secondary *BRCA* mutations that were predicted to restore *BRCA* function and homologous recombination activity.

Compensatory deleterious mutations have also been detected to confer PARP inhibitor resistance. In contrast to the homologous recombination, NHEJ only involves minor resection of DNA ends at regions of DSB [122]. *TP53* binding protein 1 (*53BP1*) maintains the balance between homologous recombination and NHEJ, and it promotes NHEJ through the inhibition of extensive DNA end-resection that is required for homologous recombination repair [123]. As such, the loss of *53BP1* function by either mutation or downregulation accelerates the *BRCA1*-independent end-resection and provides PARP inhibitor resistance [124]. It has been demonstrated that the inactivation of downstream factors of *53BP1*-mediated repair, typically *RIF1* and *REV7*, also leads to the restoration of DNA end resection, and consequently promotes homology-mediated repair [119]. In vitro studies revealed that the loss of *53BP1* function allows for the partial restoration of homologous recombination in *BRCA1*-deficient cells and counteracts sensitivity to the PARP inhibitor [125]. Heat shock protein 90 (*Hsp90*) is a crucial molecular chaperone that functions to correctly fold client proteins, and consequently prevents them from degradation by the ubiquitin-proteasome system. In vivo synergism between an *HSP90*-inhibitor (AT13387) and olaparib in PARP inhibitor resistant ovarian cancer has been described [126]. Alternatively, the evidence that acquired epigenetic changes, such as hypermethylation promoter of *BRCA1*, may restore normal *BRCA1* protein expression levels [121].

Furthermore, epigenetic silencing or accelerated protein synthesis and degradation could also lead to decreased expression of PARP enzymes, followed by PARP inhibitors resistance [127]. Another mechanism of inherent or acquired resistance is the upregulation of genes encoding p-glycoprotein efflux pumps, related to decrease intracellular drug levels [128]. In a murine breast cancer model, olaparib resulted in initial inhibited tumor growth, which is associated with an impressive increase in the expression of p-glycoprotein efflux pumps. This resistance can be reverted by the *ABCB1* inhibitors verapamil, elacridar, and tariquidar [129]. However, toxicity and lack of specificity characterize *p-glycoprotein* inhibitors.

Additional pharmacologic methods for reversing PARP inhibitor resistance have been investigated. The knockdown of cyclin-dependent kinase 12 (*CDK12*) resulted in concomitant downregulation of DNA repair proteins, and consequently the development of a “*BRCA*ness” phenotype [130]. There is in vitro evidence that pharmacological inhibition of *CDK12* with Dinaciclib reverses acquired PARP inhibitor resistance [131]. Furthermore, it has been shown that the inhibition of cell cycle regulator *WEE1* leads cells to enter the S-phase of the cell cycle, and therefore to further the accumulation of DNA DSBs in the context of HRD and PARP inhibition [132]. Overall, a combined inhibition of *CDK12* or *WEE1* could be a strategy that is recommended for overcoming homologous recombination restored PARP inhibitor resistance. 

## 14. Conclusions and Future Perspectives 

PARP inhibitors are a new class of biologic agents, which have changed the clinical management of the ovarian cancer, based upon the pre-selection characteristics of the tumors. It has been established that they have improved PFS, although a longer follow-up is required to also assess the prolongation of OS. Numerous clinical trials are ongoing, both for the currently available PARP inhibitors and those that have not yet been approved by the FDA. The analysis of *BRCA* mutational status represents a step forward to the individualized management of patients with ovarian cancer, and it should be incorporated in their diagnostic approach. Defects in homologous recombination repair seem to confer PARP inhibitors sensitivity. However, the understanding of mechanisms that contribute to clinical PARP inhibitor responses in the absence of HRD is still under investigation. The increased availability of PARP inhibitor treated specimens will potentially provide insight into novel biomarkers and acquired resistance mechanisms. It appears that treatment with PARP inhibitors is effective for patients with either germline, or somatic *BRCA1/2* mutations. The future challenge will be the optimal choice of PARP inhibitor at any given time. That demands the design of larger phase III trials, with head-to-head comparisons of them. Furthermore, PARP inhibitors have unique AEs that require further evaluation. Usually, toxicity is easily managed with supportive care and dose reduction, or modification. Further research is needed in terms of the combination of PARP inhibitor, with antiangiogenic, immunocheckpoint inhibitors, and cytotoxics as strategies for overcoming resistance mechanisms, potentiating the therapeutic efficacy, and expanding their clinical utility in non-homologous deficient tumors.

## Figures and Tables

**Table 1:** Clinical trials results for Olaparib in ovarian cancer (OC).

Study Ref.	Treatment Arms	PTS	Phase	Setting	ORR Median PFS	*p*-Value
STUDY 19 [11]	Arm1: Olaparib 400 mg BID Arm2: Placebo	265	II	1. Platinum-sensitive recurrent HGSOC, primaryperitoneal or fallopian tube cancer 2. Unselected for BRCA status 3. Maintenance treatment	1. ORR 12 vs. 4% (OR 3.36) 2. Median PFS Overall population: 8.4 vs. 4.8M (HR 0.35) *BRCA*mut: 11.2 vs. 4.3M (HR 0.18) *BRCA*wt: 7.4 vs. 5.5M (HR 0.54)	*p* = 0.12 *p* < 0.001 *p* < 0.0001 *p* = 0.0075
STUDY 42 [15]	Olaparib 400 mg BID	193	II	1. Recurrent pre-treated advanced OC, primary peritoneal or fallopian tube cancer 2. *gBRCA*1 2mut	1. ORR 34% (3+ prior regimens), 31.1% (overall) 2. Median PFS: 7.9M	
SOLO 2 [16]	Arm1: Olaparib 300 mg BID Arm2: Placebo	295	III	1. Platinum-sensitive recurrent HGSOC or HGEOC, primary peritoneal or fallopian tube cancer 2. *gBRCA1 2mut* 3. Maintenance treatment	Median PFS: 19.1 vs. 5.5M	*p* < 0.0001
SOLO 1 [17]	Arm1: Olaparib 300 mg BID Arm2: Placebo	451	III	1. Platinum sensitive after first line platinum based CT 2. g*BRCA1/2* 3. Maintenance treatment	Median PFS: NR vs. 13.8M	*p* < 0.001
Fong, P.C.; et al. [18]	Olaparib 200 mg BID	60	I		Radiological and or CA125 response 40%	
Audeh, M.W.; et al. [19]	Arm1: Olaparib 400 mg BID (*n* = 33) Arm2: Olaparib 100 mg BID (*n* = 24)	57	II	Recurrent *BRCA*-mutated OC	1. ORR 33 vs. 13% 2. Median PFS: 5.8 vs. 1.9M	
Gelmon, K.A.; et al. [20]	Olaparib 400 mg BID	64		1. Recurrent *BRCA*-mutated OC 2. Known *BRCA* status	1. ORR, *BRCA*mut: 41%, *BRCA*wt: 24% 2. Median PFS, *BRCA*mut: 7.3 M, *BRCA*wt: 6.3 M	
STUDY 12 [21]	Arm1: Olaparib 400 mg BID Arm2: Olaparib 200 mg BID Arm3: PLD 50mg m^2^	97	II	1. Recurred within 12M OC 2. Confirmed *gBRCA1 2*	1. ORR, 31 vs. 25 vs. 18% 2. Median PFS, Olaparib 200: 6.5M, Olaparib 400: 8.8M, PLD: 7.1M	*p* = 0.31 *p* < 0.66
Liu, J.F.; et al. [22]	Arm1: Olaparib 200 mg BID + cediranib 30 mg OD Arm2: Olaparib 400 mg BID	90	II	1. Platinum-sensitive recurrent HGSOC HGEOC 2. Unselected for *BRCA* status	1. ORR 79.6 vs. 47.8% (OR 4.24) 2. Median PFS 17.7 vs. 9M (HR 0.42) *BRCA*mut 19.4 vs. 16.5M (HR 0.55) *BRCA*wt 16.5 vs. 5.7M (HR 0.32)	*p* = 0.002 *p* = 0.005 *p* = 0.16 *p* = 0.008
STUDY 41 [23]	Arm1: Carboplatin AUC4 D1, paclitaxel 175 mg m^2^ D1, olaparib 200 mg BID D1-10 every 21D followed by olaparib 400 mg BID maintenance Arm2: carboplatin AUC6 D1, paclitaxel 175 mg m^2^ every 21D	162	II	Platinum sensitive recurrent HGSOC	1. ORR: 64 vs. 58% 2. Median PFS: 12.2 vs. 9.6M (HR 0.51)	*p* = 0.0012
SOLO3, NCT02282020 [24]	Arm1: Olaparib 300 mg BID Arm2: Physician’s choice CT	266	III	1. Recurrent, platinum-sensitive OC 2. *gBRCA1 2* 3. 2+ prior regimens	PFS (ongoing study)	
OReO, NCT03106987 [25]	Arm1: Olaparib 300 mg BID Arm2: Placebo		IIIb	Recurrent, platinum-sensitive OC 2. Previously treated withPARP inhibitor 3. Unselected for *BRCA* status 4. Maintenance treatment	PFS (ongoing study)	
PAOLA-1, NCT02477644 [26]	Arm1: Olaparib 300 mg BID Arm2: Placebo (in addition to bevacizumab)	612	III	1. Newly-diagnosed OC 2. PR or CR to platinum CT with bevacizumab 3. Planned bevacizumab maintenance 4. Unselected for *BRCA* Status 5. Maintenance treatment	PFS (ongoing study)	
COCOS, NCT02502266 [27]	Arm1: Olaparib Arm2: Cediranib Arm3: Olaparib + cediranib Arm4: Physician’s choice CT	680	II III	1. Recurrent, platinum-resistant OC 2. *gBRCA1 2* 3. 1-3 prior regimens	OS (ongoing study)	
NCT02446600 [28]	Arm1: Olaparib Arm2: Olaparib + cediranib Arm3: Physician’s choice CT	549	III	1. Recurrent, platinum-sensitive OC 2. *gBRCA1 2* 3. Unselected for *BRCA* status	PFS (ongoing study)	
NCT01116648 [29]	Arm1: Cediranib 30 mg + olaparib 200 mg BID Arm2: Olaparib 400 mg BID	162	I II	Recurrent Papillary-Serous Ovarian, Fallopian Tube, or Peritoneal Cancer	ORR: 44%	

Ref: reference; PTS: patients; BID: twice a day; HGSOC: high-grade serous ovarian cancer; HR: hazard ratio; OC: ovarian cancer; HGEOC: high-grade endometrioid cancer; *BRCA*mut: *BRCA* mutated; CT: chemotherapy; PFS: progression free survival; M: months; ORR: objective response rate; OR: odds ratio; g*BRCA*mut: germline *BRCA* mutated; *BRCA* wt: *BRCA* wild type; NR: not reached; PR: partial response; CR: complete response; OS: overall survival; AUC: area under the curve; D: days.

**Table 2:** Clinical trials results for Niraparib in OC.

Study Ref.	Treatment Arms	PTS	Phase	Setting	Results	*p*-Value
NOVA/ENGOT-OV16 [31]	Arm1: Niraparib 300 mg OD Arm2: Placebo	553	III	1. Recurrent, platinum-sensitive OC 2. Any *BRCA1/2* status 3. At least two prior lines of platinum-based CT with response to last platinum regimen 4. Maintenance treatment	Median PFS *gBRCA*mut: 21 vs. 5.5M, (HR 0.27) *BRCA*wt HRD(+): 12.9 vs. 3.8M, (HR 0.38) Overall non-*gBRCA*: 9.3 vs. 3.9M, (HR 0.45)	*p* < 0.0001 *p* < 0.00001 *p* < 0.0001
QUADRA [32]	Niraparib 300 mg	45	II	Platinum sensitive HRD(+) HGSOC; primary peritoneal or fallopian-tube cancer	ORR 27.5%, DCR 68.6%	
PRIMA, NCT0265501 [33]	Arm1: Niraparib 300 mg OD Arm2: Placebo	620	III	1. Newly-diagnosed OC 2. PR or CR to platinum CT 3. HRD(+) 4. Maintenance treatment	PFS (ongoing study)	

Ref: reference; PTS: patients; OD: once a day; HGSOC: high grade serous ovarian cancer; OC: ovarian cancer; *BRCA*mut: *BRCA* mutated; CT: chemotherapy; HRD(+): homologous recombination deficiency positive; HRD(−): homologous recombination deficiency negative; PFS: progression free survival; M: months; ORR: objective response rate; DCR: disease control rate; g*BRCA*mut: germline *BRCA* mutated; *BRCA*wt: *BRCA* wild type; HR: hazard ratio; PR: partial response; CR: complete response.

**Table 3:** Clinical trials results for Rucaparibin in ovarian cancer.

Study Ref.	Treatment Arms	PTS	Phase	Setting	Results	*p*-Value
STUDY 10 [35]	Rucaparib 600 mg BID	56 + 42	I II	1. Platinum-sensitive recurrent HGSOC or HGEOC, primary peritoneal or fallopian tube cancer 2. *gBRCA*mut (phase II PART 2A)	ORR: 59.5% MDR:7.8M	
ARIEL 2 PART 1 [36]	Rucaparib 600 mg BID	192	II	1. Platinum sensitive recurrent HGSOC or HGEOC, primary peritoneal or fallopian tube cancer 2. Any *BRCA* mutation status	ORR: *BRCA*mut 80%, *BRCA*wt LOH high 39%, *BRCA*wt LOH low 13% Median PFS: *BRCA*mut: 12.8M *BRCA*wt LOH High: 5.7M *BRCA*wt LOH low: 5.2M	*p* < 0.0001 *p* = 0.011
ARIEL 3 [40]	Arm1: Rucaparib 600 mg BID Arm2: Placebo	564	III	1. Platinum-sensitive recurrent HGSOC or HGEOC, primary peritoneal or fallopian tube cancer 2. Any *BRCA* mutation Status 3. ≥2 prior lines of CT 4. Maintenance treatment	Median PFS: *BRCA*mut: 16.6 vs. 5.4M HRD(+): 13.6 vs. 5.4M ITTP: 10.8 vs. 5.4M	*p* < 0.0001 *p* < 0.0001 *p* < 0.0001
ARIEL4 [41]	Arm1: rucaparib Arm2: platinum-based CT (monotherapy or doublet)	345 *	III	1. Recurrent or progressive ovarian, fallopian tube, or primary peritoneal cancer 2. ≥2 prior lines of CT	PFS (ongoing study)	

Ref: reference; PTS: patients; BID: twice a day; HGSOC: high grade serous ovarian cancer; HGEOC: high-grade endometrioid cancer; *BRCA*mut: *BRCA* mutated; CT: chemotherapy; HRD(+): homologous recombination deficiency positive; PFS: progression free survival; M: months; ORR: objective response rate; MDR: median duration of response; g*BRCA*mut: germline *BRCA* mutated; *BRCA*wt: *BRCA* wild type; LOH: loss of heterozygosity; ITTP: intention to treat population; * Estimated enrollment.

**Table 4:** Clinical trials results for Veliparib in ovarian cancer.

Study Ref.	Treatment Arms	PTS	Phase	Setting	Results	*p*-Value
GOG 280 [44]	Veliparib 400 mg BID	50	II	1. Recurrent or progressive ovarian, fallopian tube, or primary peritoneal cancer 2. ≤3 prior lines of CT 3. *gBRCA*mut	ORR 26% PFS 8.2M	
Steffensen, K.D.; et al. [46]	Veliparib 300 mg BID	16 32	I II	1. Ovarian, fallopian tube, or primary peritoneal cancer 2. Platinum-resistant or intermediate sensitive relapse 3. *gBRCA*mut	ORR 65% PFS 5.6M	
Kummar, S.; et al. [47]	Arm1: Cyclophosphamide 50 mg OD Arm2: Cyclophosphamide 50 mg OD + veliparib 60 mg OD	72	II	1. Recurrent or progressive ovarian, fallopian tube, or primary peritoneal cancer 2. ≥1 prior lines of CT 3. *gBRCA*mut	PFS: 2.3 vs. 2.41M	*p* = 0.68
GOG 3005, NCT02470585 [49]	Arm1: Carboplatin paclitaxel + placebo, followed by placebo Arm2: Carboplatin paclitaxel + veliparib, followed by placebo Arm3: Carboplatin paclitaxel + veliparib, followed by veliparib	1140	III	1. Newly-diagnosed HGSOC, fallopian tube, or primary peritoneal cancer 2. Any *BRCA* mutation status 3. Maintenance treatment	PFS (ongoing study)	
NCT01113957 [59]	Arm1: Veliparib + temozolomide Arm2: PLD	168	II	Recurrent HGSOC		

Ref: reference; PTS: patients; GOG: Gynecologic Oncology Group; BID: twice a day; HGSOC: high grade serous ovarian cancer; PLD: PEG-liposomal doxorubicin; PFS: progression free survival; M: months; ORR: objective response rate; OD: once a day; g*BRCA*mut: germline *BRCA* mutated.

**Table 5:** Clinical trials results for Talazoparib in ovarian cancer.

Study Ref.	Treatment Arms	PTS	Phase	Setting	Results Primary Objectives	Status
de Bono, J.; et al., NCT0128698 [52]	Talazoparib 1 mg OD	34	I	1. Platinum-treated HGSOC or HGEOC, primary peritoneal or fallopian tube cancer 2. *gBRCA*mut (25 34)	ORR: 42% *gBRCA*mut: ORR: 55% in platinum-sensitive ORR: 20% in platinum-resistant PFS: 36.4M	Completed
NCT0283602 [58]	Arm1: Talazoparib 1 mg OD Arm2: Talazoparib 1 mg OD + Temozolomide 37.5 mg m^2^ on D1-5	NA	II	1. Recurrent HGSOC or HGEOC, primary peritoneal or fallopian tube cancer 2. <3 prior lines of CT 3. *gBRCA*mut, or *sBRCA*mut, or HRD(+)	ORR	Ongoing
NCT02316834 POSITION trial [60]	Talazoparib 1 mg OD	30	I	1. HGSOC, primary peritoneal or fallopian tube cancer 2. No prior therapy	Basal levels and effects of talazoparib on DNA copy number, LOH and mutation, and level of RNA and protein expression in homologous recombination-related pathways before and after treatment	Ongoing

Ref: reference; PTS: patients; OD: once a day; HGSOC: high grade serous ovarian cancer; HGEOC: high-grade endometrioid cancer; CT: chemotherapy; PFS: progression free survival; M: months; ORR: objective response rate; gBRCAmut: germline *BRCA* mutated; s*BRCA*mut: somatic *BRCA* mutated; LOH: loss of heterozygosity; DNA: DeoxyriboNucleic Acid; RNA: Ribonucleic Acid; D: days; HRD(+): homologous recombination deficiency positive.

**Table 6:** United States (US) Food and Drug Administration-European Medicines Agency (FDA-EMA) approved indications for poly (ADP-ribose) polymerase (PARP) inhibitors and relevant adverse events (AEs).

	Olaparib	Niraparib	Rucaparib
Dosing	Capsules: 400 mg BID Tablets: 300 mg BID	Capsules: 300 mg BID	Tablets: 600 mg BID
Pivotal Trial	STUDY 19 [11] SOLO 2 [16] STUDY 42 [20]	NOVA TRIAL [31]	STUDY 10 [35] ARIEL 2 [36] ARIEL 3 [40]
FDA Approved Indications	2014: 1. Recurrent *gBRCA*mut, epithelial ovarian, fallopian tube, or primary peritoneal cancer, treated previously with more than 3 lines of platinum-based CT. 2. Capsules formulations. 2017: Maintenance therapy for recurrent epithelial ovarian, fallopian tube, or primary peritoneal cancer, treated previously with platinum-based CT. 2. Tablets formulations.	2017: Maintenance therapy for recurrent epithelial ovarian, fallopian tube, or primary peritoneal cancer treated previously with platinum-based CT.	2016: 1. Relapsed or progressive, platinum sensitive, *g sBRCA*mut, epithelial ovarian, fallopian tube, or primary peritoneal cancer, treated previously with 2 or more lines of platinum-based CT. 2. Patients unable to tolerate further platinum based CT 2018: Maintenance therapy for recurrent, epithelial ovarian, fallopian tube, or primary peritoneal cancer, treated previously with platinum-based CT.
EMA Approved Indications	2014: Maintenance therapy for *BRCA* mutated, platinum-sensitive relapsed epithelial ovarian, fallopian tube, or primary peritoneal cancer. 2018: 1. Maintenance therapy for platinum-sensitive relapsed epithelial ovarian, fallopian tube, or primary peritoneal cancer, regardless of *BRCA* status. 2. Tablets formulations	2017: Maintenance therapy for platinum-sensitive relapsed epithelial ovarian, fallopian tube, or primary peritoneal cancer treated previously with platinum-based CT	2018: 1. Relapsed or progressive, platinum sensitive, *g sBRCA*mut, epithelial ovarian, fallopian tube, or primary peritoneal cancer, treated previously with 2 or more lines of platinum-based CT. 2. Patients unable to tolerate further platinum based CT
AEs (all grades, ≥40% prevalence)	SOLO 2: Anemia 4%, abdominal pain 2%, intestinal obstruction 2%	Leukopenia, anemia, nausea, vomiting, metabolism nutrition, nervous system	ARIEL 2: Intestinal obstruction 2%, anemia 4%
			ARIEL 3: Anemia 4%, pyrexia 2%, vomiting 2%, intestinal obstruction 1%
AEs (grade 3 4, ≥5% prevalence)	Study 19: Fatigue 6%, anemia 5%, nausea 2%, vomiting 2%	NOVA TRIAL: Thrombocytopenia 28%, anemia 25%, neutropenia 11%, hypertension 8.2%, fatigue 8.2%, nausea 3.0%, abdominal pain 1.1%	STUDY 10: Fatigue, anemia, elevated *AST ALT*
			ARIEL 2: Anemia 45%, neutropenia 7%, elevated *ALT AST* 13%, fatigue 9%, nausea 4%, abdominal pain 2%, thrombocytopenia 2%
	SOLO 2: Anemia 18%, neutropenia 4%, fatigue 4%, nausea 3%, vomiting 3%, abdominal pain 3%, thrombocytopenia 1%		ARIEL 3: Anemia 19%, neutropenia 7%, elevated *ALT AST* 10%, fatigue 7%, nausea 4%, abdominal pain 2%, thrombocytopenia 5%
	Study 42: Fatigue 6%, anemia 19%, nausea 0.5%, vomiting 3%		
Changes in dose due to AEs	SOLO 2: Dose reductions 25%, discontinuations 11%	NOVA TRIAL: Dose reductions 66.5%, discontinuations 14.7%	ARIEL 2: Dose reductions 39%, discontinuations 9%
			ARIEL 3: Dose reductions 55%, discontinuations 13%

FDA: Food and Drug Administration; EMA: European Medicines Agency; PARP: Poly (ADP-ribose) polymerase; BID: twice a day; g*BRCA*mut: germline *BRCA* mutated; g *BRCA*: germaline *BRCA* mutated; g s*BRCA*: germline somatic *BRCA* mutated; CT: chemotherapy; AEs: adverse events; ALT: alanine aminotransferase; AST: aspartate aminotransferase.
